# Impact of Virtual Reality, Augmented Reality, and Sensor Technology in Knee Osteoarthritis Rehabilitation: A Systematic Review

**DOI:** 10.7759/cureus.79011

**Published:** 2025-02-14

**Authors:** Theodora Plavoukou, Konstantina Apostolakopoulou, Georgios Papagiannis, Dimitris Stasinopoulos, Georgios Georgoudis

**Affiliations:** 1 Department of Physiotherapy, University of West Attica (UNIWA), Athens, GRC; 2 First Department of Orthopaedic Surgery, National and Kapodistrian University of Athens School of Medicine, Athens, GRC; 3 Department of Physiotherapy, University of Peloponnese, Sparta, GRC

**Keywords:** augmented reality, knee osteoarthritis, rehabilitation, sensor technology, virtual reality, wearables

## Abstract

Knee osteoarthritis (KOA) is a progressive degenerative joint disorder that significantly impacts mobility, pain levels, and overall quality of life. Conventional rehabilitation methods, while effective, often suffer from limitations related to patient adherence, accessibility, and cost. This systematic review examines the role of virtual reality (VR), augmented reality (AR), and sensor-based technologies in KOA rehabilitation, evaluating their effectiveness in pain reduction, functional improvement, and patient engagement. A comprehensive literature search identified four randomized controlled trials (RCTs) comprising 405 participants, with an average Physiotherapy Evidence Database (PEDro) score of 6/10, indicating moderate to high methodological quality. Findings suggest that VR and AR interventions enhance rehabilitation adherence and engagement, while sensor-based systems provide real-time biofeedback, enabling personalized therapeutic adjustments. These technologies demonstrated significant improvements in pain management, muscle strength, and functional mobility. However, challenges such as high costs, limited accessibility, and the absence of standardized treatment protocols remain barriers to widespread clinical adoption. Further research should focus on long-term efficacy, cost-effectiveness, and the integration of these innovations into routine clinical practice.

## Introduction and background

Knee osteoarthritis (KOA) is a chronic, degenerative condition impacting millions globally and characterized by pain, stiffness, and functional impairment, which severely reduces patients' quality of life [1-4]. As KOA progresses, patients experience increasingly limited mobility, which often leads to further complications, such as muscle weakness, instability, and a cycle of reduced physical activity [5-8]. Given the rising prevalence of KOA, driven in part by aging populations and the obesity epidemic, the healthcare burden associated with managing and treating KOA continues to increase, underscoring the urgent need for effective, scalable rehabilitation strategies [9,10]. Traditional treatments like physical therapy and pharmacologic management play essential roles in alleviating symptoms, yet their limitations in adherence, accessibility, and cost-effectiveness make them less ideal for long-term care [3,11,12]. This has prompted the exploration of advanced technologies, including virtual reality (VR), augmented reality (AR), and sensor-based systems, which offer innovative, patient-centered solutions. VR creates immersive, simulated environments that engage patients in interactive activities, which can serve as distractions from pain and facilitate adherence to prescribed exercises [5]. Studies have shown that VR-based rehabilitation interventions reduce pain perception, enhance lower limb functionality, and improve patient engagement by providing a more enjoyable rehabilitation experience than traditional methods [2,13]. In one recent randomized controlled trial (RCT), patients with KOA who used VR during rehabilitation exercises reported significantly lower pain scores and demonstrated increased adherence to therapy [9,11]. VR’s immersive nature enables users to engage in low-impact, yet meaningful physical activity that improves functional mobility and balance, while simultaneously supporting psychological well-being by reducing anxiety and enhancing motivation [7,12]. Similarly, AR superimposes digital information into the real world, allowing patients to receive instant, real-time feedback during rehabilitation exercises, which is especially beneficial for correcting movement errors [9,12]. Unlike VR, which removes patients from the physical environment, AR blends virtual elements with real-world surroundings, thus enabling patients to follow exercises without disconnecting from their actual environment. This makes AR an ideal choice for at-home rehabilitation, where patients may not have immediate access to therapists but still benefit from interactive guidance and feedback on movement execution [9,14]. Clinical trials examining the efficacy of AR in KOA rehabilitation have shown promising results; patients using AR report faster recovery times, increased functional outcomes, and greater exercise precision, particularly compared to patients using conventional methods alone [14,15]. Wearable sensor technology adds another dimension to KOA rehabilitation by providing real-time tracking of joint movement and biomechanical data, thus enhancing personalized therapy and enabling patients and clinicians to monitor progress continuously [9,16]. These sensors can be embedded in braces or worn as patches, transmitting data on joint angles, gait patterns, and weight distribution, which is invaluable for adjusting treatment plans based on quantifiable improvements or setbacks [6,16]. A study involving sensor-embedded knee braces found that real-time feedback on movement increased adherence to rehabilitation exercises by enabling patients to monitor and modify their movements independently while receiving remote clinician support as needed [10,12]. Furthermore, the ability to track and assess progress remotely using wearable sensors makes it possible for patients to continue rehabilitation outside traditional clinical settings, thus improving accessibility and supporting long-term adherence to prescribed routines [9]. Despite the advantages offered by VR, AR, and wearable sensors, several limitations remain. Many studies highlight the short-term benefits of these technologies, such as immediate reductions in pain and improvements in function, but their long-term efficacy and cost-effectiveness remain unclear [9,16]. While VR and AR may reduce initial costs associated with frequent in-clinic visits by supporting at-home therapy, they often require substantial initial investment in devices and technical support, potentially limiting access to lower-income patients [14,15]. Moreover, adapting these advanced technologies to various patient demographics and healthcare settings remains a challenge, as many systems currently lack standardized protocols for use in rehabilitation, which complicates the implementation of these innovations into routine clinical care [15].

Despite the promising results demonstrated in individual studies, there remains considerable variability in their findings regarding the effectiveness of VR, AR, and sensor technologies in KOA rehabilitation. While some studies highlight significant improvements in pain relief, functional mobility, and adherence, others report only modest benefits, raising questions about their clinical applicability and generalizability. Moreover, the methodological heterogeneity across trials - such as differences in intervention protocols, participant characteristics, and outcome measures - complicates the interpretation of results. This inconsistency underscores the need for a comprehensive synthesis of evidence to determine the true efficacy of these technologies. By systematically analyzing and integrating positive and negative outcomes from diverse studies, this review aims to resolve uncertainties and provide a clearer understanding of the role these advanced tools play in KOA rehabilitation. This approach will ultimately guide clinicians and researchers in optimizing their use and implementation in practice.

This systematic review aims to evaluate the effectiveness of VR, AR, and sensor technologies in KOA rehabilitation, focusing on their impact on pain relief, functional mobility, and psychological well-being. By synthesizing findings from recent RCTs, this review will assess how these innovative technologies compare to traditional methods, with an emphasis on their clinical effectiveness, potential limitations, and avenues for future research. Through this review, we seek to inform the development of personalized and engaging rehabilitation protocols that could better meet the needs of KOA patients while addressing the limitations of current practices [9,15,16].

## Review

Methods

Study Protocol and Registration

This study is a systematic review based on RCTs, excluding previous systematic reviews and meta-analyses. Our results are based on data from previously published studies; therefore, no ethical approval or patient consent was required. The systematic review follows the Preferred Reporting Items for Systematic Reviews and Meta-Analyses (PRISMA) guidelines (the checklist is included in Appendix A) [17]. The a priori protocol for this review is registered with the International Prospective Register of Systematic Reviews (PROSPERO) under the registration number CRD42024614430.

Information Sources

A search for RCTs was conducted across the databases PubMed, PEDro, Cochrane, and Scopus. A total of 25 studies were identified: seven articles from PubMed, three from PEDro, five from Cochrane, and 10 from Scopus. The primary keywords used were: "virtual reality," "augmented reality," "sensor," "wearables," or" physiotherapy," and "knee osteoarthritis." After removing duplicate articles and reviewing the titles and abstracts, four studies were selected. Finally, after thoroughly reviewing the full texts, all four studies met the criteria for inclusion in this systematic review. These articles examine diverse applications of VR, AR, sensor technology, and wearables in KOA rehabilitation, focusing on outcomes such as pain management, functional improvement, and patient adherence. Only articles published in English were selected.

*Search and Eligibility Criteria* Overall search strategy: The search was conducted on September 1, 2024, using combinations of the following keywords: ("knee osteoarthritis") AND ("virtual reality" OR "augmented reality" OR "sensor" OR "wearables" AND ("physical therapy"). Detailed search strategies for each database, including PubMed, PEDro, Cochrane, and Scopus, are documented in the Rayyan® platform (Qatar Computing Research Institute, Qatar). Additionally, reference lists from identified articles were manually screened to capture relevant studies not found in the initial electronic searches. Inclusion and exclusion criteria were defined based on the PICO (Population, Intervention, Comparison, Outcome) framework [18,19].

Interventions: The interventions in the studies included in this systematic review involved the use of VR, AR, and sensor technologies for the rehabilitation of patients with KOA. VR and AR interventions comprised rehabilitation programs with interactive exercises such as balance training, mobility exercises, and therapy via virtual simulations. Sensor technology was employed for real-time feedback on patient movements, enabling more precise and personalized rehabilitation. In most cases, the intervention groups received support through these technologies, often alongside conventional treatments such as physiotherapist-guided programs or home-based exercise routines. Control groups underwent traditional rehabilitation methods, including clinic-based physiotherapy or conservative care without the use of VR, AR, or sensors.

Outcome measures: The primary outcomes in the studies were pain, kinesiophobia, disability, balance, and depression. Pain was measured using the Visual Analog Scale (VAS), Numeric Rating Scale (NRS), and Western Ontario and McMaster Universities Osteoarthritis Index (WOMAC) pain subscale. Kinesiophobia was assessed with the American Shoulder and Elbow Surgeons (ASES) questionnaire and the Pain-Related Anxiety and Avoidance Scale (PASC). Disability was evaluated using WOMAC and Knee Injury and Osteoarthritis Outcome Score (KOOS) functional subscales. Balance was gauged through the Lequesne Index and muscular strength tests. Depression levels were measured with the Positive and Negative Affect Schedule (PANAS) and the Arthritis Impact Measurement Scales 2 (AIMS 2) psychological subscales. These validated tools provided quantitative insights into VR, AR, and sensor-enhanced rehabilitation effectiveness for chronic musculoskeletal disorders.

Inclusion criteria: The inclusion criteria included RCTs with no restrictions on publication year, studies involving adult patients diagnosed with KOA, interventions assessing the impact of VR, AR, or sensor-based technologies as standalone therapies or in comparison with conventional rehabilitation methods, and studies evaluating clinically relevant outcomes such as pain, kinesiophobia, disability, balance, or depression.

Exclusion criteria: The exclusion criteria were as follows: studies involving patients under 18 years of age, studies without described protocols, incomplete articles, systematic reviews or meta-analyses, case studies, and articles published in languages other than English. *Data Extraction and Management*

The Rayyan platform was utilized for managing article selection and removing duplicate entries. In the first phase, two independent reviewers conducted a blind screening of article titles and abstracts to exclude irrelevant studies; the disagreements between independent reviewers were resolved through arbitration by a third reviewer. In the subsequent phase, the reviewers assessed full-text articles based on the predefined inclusion and exclusion criteria. Data extraction was performed once the appropriate RCTs were identified.

Risk of Bias

In this systematic review, the Physiotherapy Evidence Database (PEDro) scale and the Modified Downs and Black checklist were employed to assess the risk of bias in each study. Two independent reviewers applied these tools and scored each included RCT accordingly.

Results

Search and Selection

**Figure 1:**
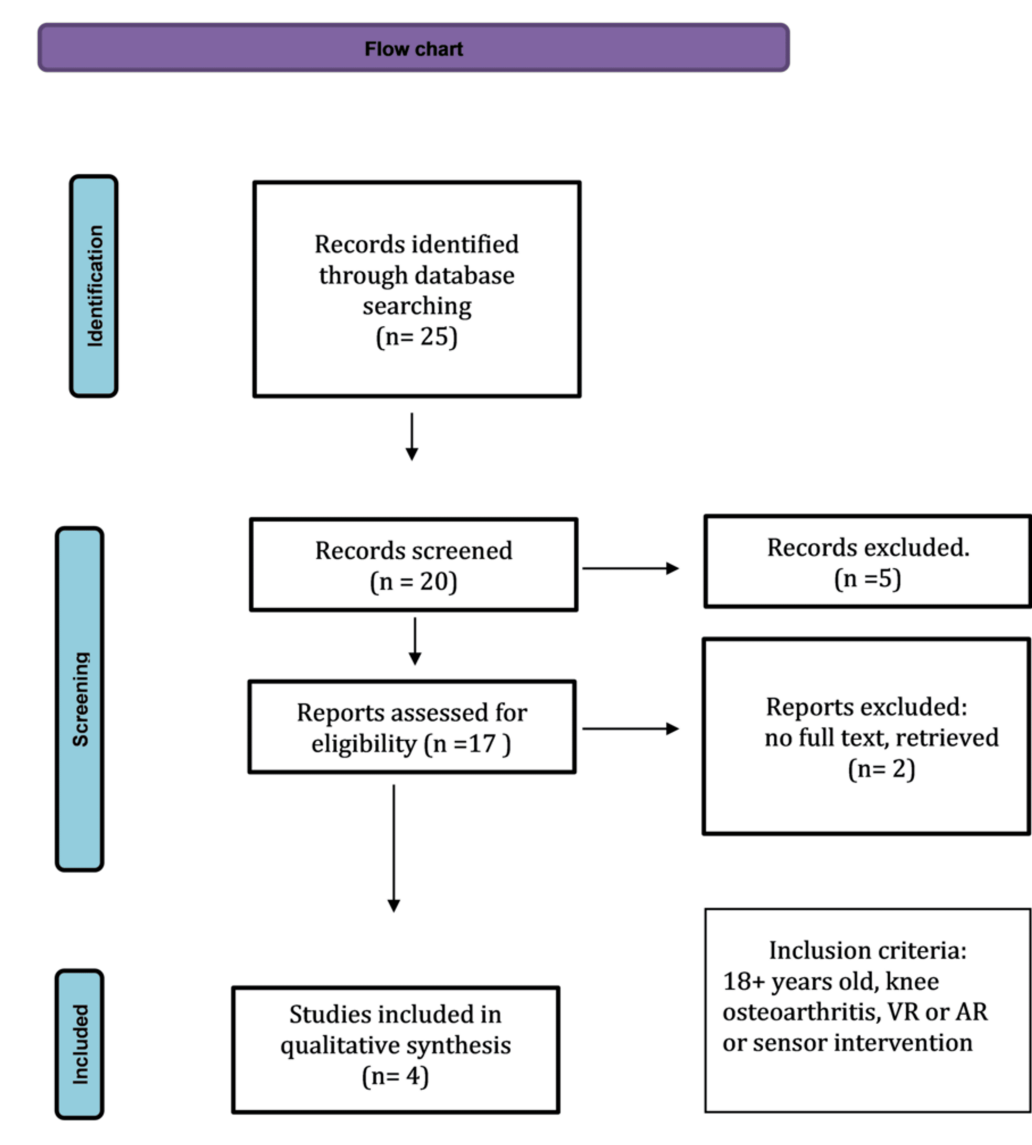
Preferred Reporting Items for Systematic Reviews and Meta-Analyses (PRISMA) flow diagram VR: virtual reality; AR: augmented reality

In total, four studies were included, with a combined participant number of 405 patients with KOA. The flow chart is described in Figure 1.

Intervention Protocols

The interventions in the four included RCTs were as follows: (1) telerehabilitation compared to electrotherapy and home exercise [20], (2) telerehabilitation combined with an online Patient-Centered Self-Management (PCST) rehabilitation program compared to usual care [21], (3) telerehabilitation with an eight-week pain management coaching (PainCOACH) program compared to rehabilitation without PainCOACH [13], and (4) rehabilitation programs incorporating VR tools such as Nintendo Wii Fit and Microsoft Xbox Kinect compared to conventional physiotherapy programs [22].

Risk of Bias

In order to score the PEDro scale, the reviewers applied the 11 criteria included in the tool and categorized the studies based on their total scores: 0-3 were considered "POOR," 4-5 "FAIR," 6-8 "GOOD," and 9-10 "EXCELLENT." Any disagreements between the two reviewers were resolved through discussion or with the help of a third evaluator. The average score of the PEDro scale across the four studies was 6/10, indicating good quality. More specifically, one study scored 8/10 [23], which is classified as excellent quality. Two studies scored 6/10 [20,22], both of which are considered good quality according to the scale. However, one study scored only 4/10 [21], which classifies it as a fair-quality study. Based on the Downs and Black checklist, three studies were classified as "FAIR" quality [20-22], and one study was classified as "GOOD" quality [13]. This indicates that the majority of the studies demonstrate moderate methodological quality, while one study shows a high level of methodological rigor. These classifications are summarized in Tables 1-2.

**Table 1:** PEDro scale Mean Score = 6/10 PEDro: Physiotherapy Evidence Database; RCT: randomized controlled trial

PEDro Scale Criteria												
RCT	1	2	3	4	5	6	7	8	9	10	11	SCORE
Azma et al., 2017 [20]	+	+	-	+	-	-	-	+	+	+	+	6/10
Lawford et al., 2018 [21]	+	-	-	-	-	-	-	+	+	+	+	4/10
Cyrillo and Greve, 2018 [22]	+	+	+	+	-	-	-	-	-	+	+	6/10
Rini et al., 2015 [13]	+	+	+	+	-	+	-	+	+	+	+	8/10

**Table 2:** Downs and Black scores RED = POOR (≤14); YELLOW = FAIR (15-19); BLUE = GOOD (20-25); GREEN = EXCELLENT (26-28)

RCT	Downs and Black Score	Quality of Study	Color-Coding
Azma et al., 2017 [20]	18/28	FAIR	YELLOW
Lawford et al., 2018 [21]	16/28	FAIR	YELLOW
Cyrillo and Greve, 2018 [22]	16/28	FAIR	YELLOW
Rini et al., 2015 [13]	22/28	GOOD	BLUE

Study Characteristics

The baseline characteristics of the included studies in this systematic review are summarized in Table 3. The studies involved participants with KOA, with sample sizes ranging from 54 to 148 individuals. The mean age of the participants varied between 37 and 72 years, ensuring a diverse representation of age groups. All studies included both male and female participants, reflecting a balanced gender distribution. Geographically, the studies were conducted in a variety of countries, including Brazil, Iran, Australia, the USA, Turkey, and Nigeria, providing a broad international perspective. These characteristics highlight the diversity of the included studies in terms of demographic and geographical contexts, strengthening the generalizability of the findings.

**Table 3:** Summary of studies on telerehabilitation in knee osteoarthritis VAS: Visual Analog Scale; PainCOACH: Pain management coaching; WOMAC: Western Ontario and McMaster Universities Osteoarthritis Index; KOOS: Knee Injury and Osteoarthritis Outcome Score; NRS: Numeric Rating Scale; AIMS 2: Arthritis Impact Measurement Scales 2; ASES: American Shoulder and Elbow Surgeons; PASC: Pain-Related Anxiety and Avoidance Scale; PANAS: Positive and Negative Affect Schedule

Study Title	Author(s) and Year	Sample Size and Condition	Assessment Tools	Method	Outcome
Efficacy of tele-rehabilitation compared with office-based physical therapy in patients with knee osteoarthritis: a randomized clinical trial	Azma et al., 2017 [20]	N=54 knee osteoarthritis	VAS, KOOS, WOMAC	Telerehabilitation group: 3 times/week for 6 weeks; Control group: 3 times/week in the clinic for 6 weeks	Significant improvement in both groups on all scales, no significant differences between groups.
Moderators of effects of internet-delivered exercise and pain coping skills training for people with knee osteoarthritis: exploratory analysis of the impact randomized controlled trial	Lawford et al., 2018 [21]	N=148 knee osteoarthritis	NRS, WOMAC	Intervention: Exercise and pain management training via Skype and online; Control: Educational materials only.	Significant pain reduction in employed individuals of the intervention group compared to the control.
The effects of virtual reality on the rehabilitation of patients with knee OA: a randomized controlled clinical trial	Cyrillo and Greve, 2018 [22]	Ν=90 patients (50-70 years old)	WOMAC and Lequesne questionnaires, pain and muscular strength evaluation	Participants divided into 3 groups (control, Wii, Kinect), performing conventional or VR-enhanced physiotherapy	Significant improvement in pain and strength across all groups; VR groups showed better functional outcomes in WOMAC scores.
Automated internet-based pain coping skills training to manage osteoarthritis pain: a randomized controlled trial	Rini et al., 2015 [13]	N=113 knee osteoarthritis	AIMS 2, ASES, PASC, PANAS	PainCOACH program for 8 weeks. Control group: same program without PainCOACH access.	Significant improvement in self-efficacy and pain reduction in women from the intervention group.

Discussions

This systematic review sheds light on the innovative use of VR, AR, and sensor technologies in the rehabilitation of patients with KOA. These technologies represent a significant shift from traditional rehabilitation methods, providing new avenues for pain management, functional improvement, and patient engagement.

The results of the included studies were analyzed based on the key parameters assessed: pain, kinesiophobia, functional disability, balance, and depression levels, using specific evaluation tools in each intervention. Pain reduction was evident across all studies, with tools such as VAS, NRS, and WOMAC demonstrating statistically significant improvements in both intervention and control groups. For instance, Azma et al. (2017) [20] and Cyrillo and Greve (2018) [22] highlighted that VR and telerehabilitation technologies produced comparable improvements to conventional physical therapy, with VR showing slightly better functional outcomes.

Kinesiophobia was not explicitly measured as a primary outcome in most studies, but improvements in pain and enhanced self-efficacy, assessed using tools like ASES, suggested indirect benefits. These improvements helped patients build confidence in managing their symptoms. Similarly, functional disability, evaluated through WOMAC and KOOS, showed significant enhancements in mobility and functionality, especially in long-term interventions incorporating pain education or VR programs. The PainCOACH program further demonstrated increased self-efficacy in women, along with reduced activity-related limitations.

Balance and neuromuscular control were positively impacted by VR-based therapies, as evidenced by tools like the Lequesne Index and muscular strength assessments. These interventions enhanced stability and strength, emphasizing the effectiveness of VR in promoting functional recovery. Depression levels were indirectly addressed using tools such as AIMS 2, PASC, and PANAS, which captured psychological well-being and emotional states. VR/AR interventions contributed to better psychological outcomes by reducing pain and improving functionality. These findings underscore the broad potential of VR and AR technologies to support both physical and psychological rehabilitation, advocating their integration into personalized protocols for chronic musculoskeletal disorders.

The included studies demonstrated that VR tools, such as Nintendo Wii and Microsoft Kinect, significantly enhance patient participation by offering immersive and interactive environments. The study by Cyrillo and Greve (2018) [22] highlighted that patients using VR-enhanced physiotherapy achieved better WOMAC functional scores compared to conventional physiotherapy. These findings underscore VR's motivational advantages, particularly for patients who may struggle with adherence to monotonous traditional programs. Furthermore, VR's ability to simulate real-life activities allows for targeted rehabilitation that aligns with patients' daily functional needs.

AR, though less prominently featured in the included studies, holds immense potential for rehabilitation. By overlaying virtual instructions in real-world settings, AR provides real-time feedback and facilitates precise movement corrections. This capability makes AR particularly effective in home-based rehabilitation, offering patients autonomy while ensuring they maintain proper exercise techniques. Future studies should explore AR's applications in KOA rehabilitation to fully understand its benefits and limitations.

Sensor technologies are revolutionizing the way rehabilitation outcomes are monitored and managed. Wearable sensors provide real-time data on joint angles, movement patterns, and weight distribution, enabling healthcare professionals to personalize interventions based on measurable progress. These devices empower patients to self-monitor their rehabilitation, fostering a sense of ownership and accountability. The studies reviewed highlight the effectiveness of sensors in extending the reach of healthcare providers through remote monitoring, a feature that has gained prominence in the post-pandemic healthcare landscape.

Despite these promising advancements, there are notable challenges and limitations. The methodological quality of the included studies, as assessed by the PEDro and Downs and Black scales, varied significantly. While Rini et al. (2015) [13] received a "GOOD" rating, other studies were classified as "FAIR," often due to the lack of blinding and limited long-term follow-up. These methodological issues highlight the need for rigorously designed trials with larger sample sizes and standardized protocols to strengthen the evidence base.

Another critical issue is accessibility. The initial costs and technological requirements of VR, AR, and sensor systems may pose barriers, particularly in low-resource settings. Additionally, the absence of universal guidelines for integrating these technologies into rehabilitation practice hinders their widespread adoption. Addressing these barriers will require collaboration among researchers, healthcare providers, and technology developers.

The findings of this review highlight the potential of VR, AR, and sensor technologies as valuable tools in KOA rehabilitation. By complementing traditional physiotherapy, these innovations offer scalable and patient-centered solutions that enhance outcomes across various domains. However, to maximize their utility, future research must focus on long-term evaluations, cost-effectiveness analyses, and the development of comprehensive implementation frameworks.

## Conclusions

The integration of VR, AR, and sensor technologies in KOA rehabilitation represents an innovative approach with significant benefits for both patients and healthcare professionals. Findings from this systematic review confirm that these technologies contribute to pain reduction, improved muscle strength, and enhanced functional mobility, while also promoting patient adherence through interactive and personalized rehabilitation programs. Additionally, real-time data collection and remote monitoring expand access to physiotherapy services, enabling individualized interventions and improved treatment outcomes.

However, despite these advantages, challenges remain, including high costs, technological accessibility, and the lack of standardized protocols for clinical implementation. Future research should focus on evaluating the long-term effectiveness and cost-benefit ratio of these interventions to ensure their sustainable and widespread adoption. Ongoing education and training for healthcare professionals in digital rehabilitation technologies will be crucial for maximizing their potential, ultimately improving the quality of physiotherapy care and reshaping rehabilitation strategies for KOA.

## Appendices

Appendix A 

**Table 4:** Preferred Reporting Items for Systematic Reviews and Meta-Analyses (PRISMA) 2020 checklist Page MJ, McKenzie JE, Bossuyt PM, et al. The PRISMA 2020 statement: an updated guideline for reporting systematic reviews. BMJ 2021;372:n71. 10.1136/bmj.n71

Section and Topic	Item #	Checklist item	Location where item is reported
TITLE			
Title	1	Identify the report as a systematic review.	Title page
ABSTRACT			
Abstract	2	See the PRISMA 2020 for Abstracts checklist.	Abstract
INTRODUCTION			
Rationale	3	Describe the rationale for the review in the context of existing knowledge.	Introduction
Objectives	4	Provide an explicit statement of the objective(s) or question(s) the review addresses.	Introduction
METHODS			
Eligibility criteria	5	Specify the inclusion and exclusion criteria for the review and how studies were grouped for the syntheses.	Methods – Search and Eligibility Criteria
Information sources	6	Specify all databases, registers, websites, organisations, reference lists and other sources searched or consulted to identify studies. Specify the date when each source was last searched or consulted.	Methods – Information Sources
Search strategy	7	Present the full search strategies for all databases, registers and websites, including any filters and limits used.	Methods – Overall Search Strategy
Selection process	8	Specify the methods used to decide whether a study met the inclusion criteria of the review, including how many reviewers screened each record and each report retrieved, whether they worked independently, and if applicable, details of automation tools used in the process.	Methods – Data Extraction and Management
Data collection process	9	Specify the methods used to collect data from reports, including how many reviewers collected data from each report, whether they worked independently, any processes for obtaining or confirming data from study investigators, and if applicable, details of automation tools used in the process.	Methods – Data Extraction and Management
Data items	10a	List and define all outcomes for which data were sought. Specify whether all results that were compatible with each outcome domain in each study were sought (e.g. for all measures, time points, analyses), and if not, the methods used to decide which results to collect.	Methods – Outcome Measures
	10b	List and define all other variables for which data were sought (e.g. participant and intervention characteristics, funding sources). Describe any assumptions made about any missing or unclear information.	Methods - Interventions
Study risk of bias assessment	11	Specify the methods used to assess risk of bias in the included studies, including details of the tool(s) used, how many reviewers assessed each study and whether they worked independently, and if applicable, details of automation tools used in the process.	Methods – Risk of Bias
Effect measures	12	Specify for each outcome the effect measure(s) (e.g. risk ratio, mean difference) used in the synthesis or presentation of results.	Methods – Outcome Measures
Synthesis methods	13a	Describe the processes used to decide which studies were eligible for each synthesis (e.g. tabulating the study intervention characteristics and comparing against the planned groups for each synthesis (item #5)).	Methods - Interventions
	13b	Describe any methods required to prepare the data for presentation or synthesis, such as handling of missing summary statistics, or data conversions.	Methods – Data Extraction and Management
	13c	Describe any methods used to tabulate or visually display results of individual studies and syntheses.	Results – Table 1, Table 2, Table 3
	13d	Describe any methods used to synthesize results and provide a rationale for the choice(s). If meta-analysis was performed, describe the model(s), method(s) to identify the presence and extent of statistical heterogeneity, and software package(s) used.	Results – Intervention Protocols
	13e	Describe any methods used to explore possible causes of heterogeneity among study results (e.g. subgroup analysis, meta-regression).	Discussion – Study Limitations
	13f	Describe any sensitivity analyses conducted to assess robustness of the synthesized results.	Discussion – Limitations and Future Research
Reporting bias assessment	14	Describe any methods used to assess risk of bias due to missing results in a synthesis (arising from reporting biases).	Methods – Risk of Bias
Certainty assessment	15	Describe any methods used to assess certainty (or confidence) in the body of evidence for an outcome.	Discussion – Methodological Quality
RESULTS			
Study selection	16a	Describe the results of the search and selection process, from the number of records identified in the search to the number of studies included in the review, ideally using a flow diagram.	Results – Figure 1
	16b	Cite studies that might appear to meet the inclusion criteria, but which were excluded, and explain why they were excluded.	Results – Search and Selection
Study characteristics	17	Cite each included study and present its characteristics.	Results – Table 3
Risk of bias in studies	18	Present assessments of risk of bias for each included study.	Results – Table 1, Table 2
Results of individual studies	19	For all outcomes, present, for each study: (a) summary statistics for each group (where appropriate) and (b) an effect estimate and its precision (e.g. confidence/credible interval), ideally using structured tables or plots.	Results – Study Characteristics
Results of syntheses	20a	For each synthesis, briefly summarise the characteristics and risk of bias among contributing studies.	Results – Risk of Bias
	20b	Present results of all statistical syntheses conducted. If meta-analysis was done, present for each the summary estimate and its precision (e.g. confidence/credible interval) and measures of statistical heterogeneity. If comparing groups, describe the direction of the effect.	Results – Intervention Protocols
	20c	Present results of all investigations of possible causes of heterogeneity among study results.	Discussion – Study Limitations
	20d	Present results of all sensitivity analyses conducted to assess the robustness of the synthesized results.	Discussion – Limitations and Future Research
Reporting biases	21	Present assessments of risk of bias due to missing results (arising from reporting biases) for each synthesis assessed.	Discussion – Methodological Quality
Certainty of evidence	22	Present assessments of certainty (or confidence) in the body of evidence for each outcome assessed.	Discussion – Limitations and Future Research
DISCUSSION			
Discussion	23a	Provide a general interpretation of the results in the context of other evidence.	Discussion
	23b	Discuss any limitations of the evidence included in the review.	Discussion – Study Limitations
	23c	Discuss any limitations of the review processes used.	Discussion – Limitations and Future Research
	23d	Discuss implications of the results for practice, policy, and future research.	Conclusion
OTHER INFORMATION			
Registration and protocol	24a	Provide registration information for the review, including register name and registration number, or state that the review was not registered.	Methods – Study Protocol and Registration
	24b	Indicate where the review protocol can be accessed, or state that a protocol was not prepared.	Methods – Study Protocol and Registration
	24c	Describe and explain any amendments to information provided at registration or in the protocol.	Methods – Study Protocol and Registration
Support	25	Describe sources of financial or non-financial support for the review, and the role of the funders or sponsors in the review.	Acknowledgements
Competing interests	26	Declare any competing interests of review authors.	Conflict of Interest
Availability of data, code and other materials	27	Report which of the following are publicly available and where they can be found: template data collection forms; data extracted from included studies; data used for all analyses; analytic code; any other materials used in the review.	Supplementary Materials
Section and Topic	Item #	Checklist item	Location where item is reported
TITLE			
Title	1	Identify the report as a systematic review.	Title page
ABSTRACT			
Abstract	2	See the PRISMA 2020 for Abstracts checklist.	Abstract
INTRODUCTION			
Rationale	3	Describe the rationale for the review in the context of existing knowledge.	Introduction
Objectives	4	Provide an explicit statement of the objective(s) or question(s) the review addresses.	Introduction
METHODS			
Eligibility criteria	5	Specify the inclusion and exclusion criteria for the review and how studies were grouped for the syntheses.	Methods – Search and Eligibility Criteria
Information sources	6	Specify all databases, registers, websites, organisations, reference lists and other sources searched or consulted to identify studies. Specify the date when each source was last searched or consulted.	Methods – Information Sources
Search strategy	7	Present the full search strategies for all databases, registers and websites, including any filters and limits used.	Methods – Overall Search Strategy
Selection process	8	Specify the methods used to decide whether a study met the inclusion criteria of the review, including how many reviewers screened each record and each report retrieved, whether they worked independently, and if applicable, details of automation tools used in the process.	Methods – Data Extraction and Management
Data collection process	9	Specify the methods used to collect data from reports, including how many reviewers collected data from each report, whether they worked independently, any processes for obtaining or confirming data from study investigators, and if applicable, details of automation tools used in the process.	Methods – Data Extraction and Management
Data items	10a	List and define all outcomes for which data were sought. Specify whether all results that were compatible with each outcome domain in each study were sought (e.g. for all measures, time points, analyses), and if not, the methods used to decide which results to collect.	Methods – Outcome Measures
	10b	List and define all other variables for which data were sought (e.g. participant and intervention characteristics, funding sources). Describe any assumptions made about any missing or unclear information.	Methods - Interventions
Study risk of bias assessment	11	Specify the methods used to assess risk of bias in the included studies, including details of the tool(s) used, how many reviewers assessed each study and whether they worked independently, and if applicable, details of automation tools used in the process.	Methods – Risk of Bias
Effect measures	12	Specify for each outcome the effect measure(s) (e.g. risk ratio, mean difference) used in the synthesis or presentation of results.	Methods – Outcome Measures
Synthesis methods	13a	Describe the processes used to decide which studies were eligible for each synthesis (e.g. tabulating the study intervention characteristics and comparing against the planned groups for each synthesis (item #5)).	Methods - Interventions
	13b	Describe any methods required to prepare the data for presentation or synthesis, such as handling of missing summary statistics, or data conversions.	Methods – Data Extraction and Management
	13c	Describe any methods used to tabulate or visually display results of individual studies and syntheses.	Results – Table 1, Table 2, Table 3
	13d	Describe any methods used to synthesize results and provide a rationale for the choice(s). If meta-analysis was performed, describe the model(s), method(s) to identify the presence and extent of statistical heterogeneity, and software package(s) used.	Results – Intervention Protocols
	13e	Describe any methods used to explore possible causes of heterogeneity among study results (e.g. subgroup analysis, meta-regression).	Discussion – Study Limitations
	13f	Describe any sensitivity analyses conducted to assess robustness of the synthesized results.	Discussion – Limitations and Future Research
Reporting bias assessment	14	Describe any methods used to assess risk of bias due to missing results in a synthesis (arising from reporting biases).	Methods – Risk of Bias
Certainty assessment	15	Describe any methods used to assess certainty (or confidence) in the body of evidence for an outcome.	Discussion – Methodological Quality
RESULTS			
Study selection	16a	Describe the results of the search and selection process, from the number of records identified in the search to the number of studies included in the review, ideally using a flow diagram.	Results – Figure 1
	16b	Cite studies that might appear to meet the inclusion criteria, but which were excluded, and explain why they were excluded.	Results – Search and Selection
Study characteristics	17	Cite each included study and present its characteristics.	Results – Table 3
Risk of bias in studies	18	Present assessments of risk of bias for each included study.	Results – Table 1, Table 2
Results of individual studies	19	For all outcomes, present, for each study: (a) summary statistics for each group (where appropriate) and (b) an effect estimate and its precision (e.g. confidence/credible interval), ideally using structured tables or plots.	Results – Study Characteristics
Results of syntheses	20a	For each synthesis, briefly summarise the characteristics and risk of bias among contributing studies.	Results – Risk of Bias
	20b	Present results of all statistical syntheses conducted. If meta-analysis was done, present for each the summary estimate and its precision (e.g. confidence/credible interval) and measures of statistical heterogeneity. If comparing groups, describe the direction of the effect.	Results – Intervention Protocols
	20c	Present results of all investigations of possible causes of heterogeneity among study results.	Discussion – Study Limitations
	20d	Present results of all sensitivity analyses conducted to assess the robustness of the synthesized results.	Discussion – Limitations and Future Research
Reporting biases	21	Present assessments of risk of bias due to missing results (arising from reporting biases) for each synthesis assessed.	Discussion – Methodological Quality
Certainty of evidence	22	Present assessments of certainty (or confidence) in the body of evidence for each outcome assessed.	Discussion – Limitations and Future Research
DISCUSSION			
Discussion	23a	Provide a general interpretation of the results in the context of other evidence.	Discussion
	23b	Discuss any limitations of the evidence included in the review.	Discussion – Study Limitations
	23c	Discuss any limitations of the review processes used.	Discussion – Limitations and Future Research
	23d	Discuss implications of the results for practice, policy, and future research.	Conclusion
OTHER INFORMATION			
Registration and protocol	24a	Provide registration information for the review, including register name and registration number, or state that the review was not registered.	Methods – Study Protocol and Registration
	24b	Indicate where the review protocol can be accessed, or state that a protocol was not prepared.	Methods – Study Protocol and Registration
	24c	Describe and explain any amendments to information provided at registration or in the protocol.	Methods – Study Protocol and Registration
Support	25	Describe sources of financial or non-financial support for the review, and the role of the funders or sponsors in the review.	Acknowledgements
Competing interests	26	Declare any competing interests of review authors.	Conflict of Interest
Availability of data, code and other materials	27	Report which of the following are publicly available and where they can be found: template data collection forms; data extracted from included studies; data used for all analyses; analytic code; any other materials used in the review.	Supplementary Materials

## References

[1] Cui A, Li H, Wang D, Zhong J, Chen Y, Lu H (2020). Global, regional prevalence, incidence and risk factors of knee osteoarthritis in population-based studies. EClinicalMedicine.

[2] Abramoff B, Caldera FE (2020). Osteoarthritis: pathology, diagnosis, and treatment options. Med Clin North Am.

[3] Naeemabadi Mr, Fazlali H, Najafi S, Dinesen B, Hansen J (2020). Telerehabilitation for patients with knee osteoarthritis: a focused review of technologies and teleservices. JMIR Biomed Eng.

[4] Odole AC, Ojo OD (2013). A telephone-based physiotherapy intervention for patients with osteoarthritis of the knee. Int J Telerehabil.

[5] Xia B, Di Chen, Zhang J, Hu S, Jin H, Tong P (2014). Osteoarthritis pathogenesis: a review of molecular mechanisms. Calcif Tissue Int.

[6] Arntz A, Weber F, Handgraaf M (2023). Technologies in home-based digital rehabilitation: scoping review. JMIR Rehabil Assist Technol.

[7] Kolasinski SL, Neogi T, Hochberg MC (2020). 2019 American College of Rheumatology/Arthritis Foundation guideline for the management of osteoarthritis of the hand, hip, and knee. Arthritis Rheumatol.

[8] Xie SH, Wang Q, Wang LQ, Wang L, Song KP, He CQ (2021). Effect of internet-based rehabilitation programs on improvement of pain and physical function in patients with knee osteoarthritis: systematic review and meta-analysis of randomized controlled trials. J Med Internet Res.

[9] Wei W, Tang H, Luo Y (2024). Efficacy of virtual reality exercise in knee osteoarthritis rehabilitation: a systematic review and meta-analysis. Front Physiol.

[10] Inan OT, Whittingslow DC, Teague CN (2018). Wearable knee health system employing novel physiological biomarkers. J Appl Physiol (1985).

[11] Xiang W, Wang JY, Ji BJ, Li LJ, Xiang H (2023). Effectiveness of different telerehabilitation strategies on pain and physical function in patients with knee osteoarthritis: systematic review and meta-analysis. J Med Internet Res.

[12] Byra J, Czernicki K (2020). The effectiveness of virtual reality rehabilitation in patients with knee and hip osteoarthritis. J Clin Med.

[13] Rini C, Porter LS, Somers TJ (2015). Automated internet-based pain coping skills training to manage osteoarthritis pain: a randomized controlled trial. Pain.

[14] Desai K, Bahirat K, Ramalingam S, Prabhakaran B, Annaswamy M, Makris UE (2016). Augmented reality-based exergames for rehabilitation. Proc ACM CHI Conf Hum Factors Comput Syst.

[15] Perna A, Proietti L (2023). Editorial on: musculoskeletal rehabilitation: current challenges and new perspectives. J Clin Med.

[16] Berton A, Longo UG, Candela V (2020). Virtual reality, augmented reality, gamification, and telerehabilitation: psychological impact on orthopedic patients' rehabilitation. J Clin Med.

[17] Moher D, Liberati A, Tetzlaff J, Altman DG (2009). Preferred reporting items for systematic reviews and meta-analyses: the PRISMA statement. BMJ.

[18] Booth A, Clarke M, Dooley G, Ghersi D, Moher D, Petticrew M, Stewart L (2012). The nuts and bolts of PROSPERO: an international prospective register of systematic reviews. Syst Rev.

[19] Guyatt GH, Oxman AD, Kunz R (2011). GRADE guidelines: 2. Framing the question and deciding on important outcomes. J Clin Epidemiol.

[20] Azma K, RezaSoltani Z, Rezaeimoghaddam F, Dadarkhah A, Mohsenolhosseini S (2018). Efficacy of tele-rehabilitation compared with office-based physical therapy in patients with knee osteoarthritis: a randomized clinical trial. J Telemed Telecare.

[21] Lawford BJ, Hinman RS, Kasza J, Nelligan R, Keefe F, Rini C, Bennell KL (2018). Moderators of effects of internet-delivered exercise and pain coping skills training for people with knee osteoarthritis: exploratory analysis of the impact randomized controlled trial. J Med Internet Res.

[22] Cyrillo F, Greve J (2018). The effects of virtual reality on the rehabilitation of patients with knee OA: a randomized controlled clinical trial. Ann Phys Rehabil Med.

[23] Tore NG, Oskay D, Haznedaroglu S (2023). The quality of physiotherapy and rehabilitation program and the effect of telerehabilitation on patients with knee osteoarthritis. Clin Rheumatol.

